# Prediction Models of Early Childhood Caries Based on Machine Learning Algorithms

**DOI:** 10.3390/ijerph18168613

**Published:** 2021-08-15

**Authors:** You-Hyun Park, Sung-Hwa Kim, Yoon-Young Choi

**Affiliations:** 1 Department of Biostatistics, Yonsei University Wonju College of Medicine, Wonju 26426, Korea; dbgus6175@yonsei.ac.kr (Y.-H.P.); juniver1057@naver.com (S.-H.K.); 2 Artificial Intelligence Big Data Medical Center, Yonsei University Wonju College of Medicine, Wonju 26426, Korea

**Keywords:** early childhood caries, Korea National Health and Nutrition Survey, machine learning, prediction

## Abstract

In this study, we developed machine learning-based prediction models for early childhood caries and compared their performances with the traditional regression model. We analyzed the data of 4195 children aged 1–5 years from the Korea National Health and Nutrition Examination Survey data (2007–2018). Moreover, we developed prediction models using the XGBoost (version 1.3.1), random forest, and LightGBM (version 3.1.1) algorithms in addition to logistic regression. Two different methods were applied for variable selection, including a regression-based backward elimination and a random forest-based permutation importance classifier. We compared the area under the receiver operating characteristic (AUROC) values and misclassification rates of the different models and observed that all four prediction models had AUROC values ranging between 0.774 and 0.785. Furthermore, no significant difference was observed between the AUROC values of the four models. Based on the results, we can confirm that both traditional logistic regression and ML-based models can show favorable performance and can be used to predict early childhood caries, identify ECC high-risk groups, and implement active preventive treatments. However, further research is essential to improving the performance of the prediction model using recent methods, such as deep learning.

## 1. Introduction

Early childhood caries (ECC) refers to the presence of one or more decayed, missing, or filled tooth surfaces in a child under six years of age [1] and can cause severe pain, tooth loss, low self-esteem, low school performance, sleep disturbance, and reduce quality of life [2,3,4]. Considering oral diseases can have adverse effects on the development of permanent teeth, a healthy oral environment during early childhood is essential for determining one’s oral health for a lifetime [5]. Therefore, the prevention of ECC is highly necessary for children to maintain their oral health, a healthy life, and reduced medical costs [6]. Although a sharp decline in ECC was observed in Korea until the early 2000s due to the efforts of academia and clinicians, there has been an increase since 2016 [7]. Based on previous studies, risk factors such as socioeconomic factors [8], parents’ knowledge on oral health [9], type of feeding [10], time at which weaning food was introduced [11], sugar consumption [8], allergic disease [12], and overweightness [13] have been identified for the early life prediction of ECC. The prediction of caries risk based on these risk factors is considered a cornerstone of clinical decision making and disease prevention in individual patients [14] and using comprehensive and standardized tools is recommended [15]. While many have attempted to evaluate the risk of dental caries in early childhood [16,17,18], none have developed a prediction model that uses large-scale, representative survey data from the Korea National Health and Nutrition Examination Survey (KNHANES).

In recent years, machine learning (ML) is being widely used in medicine for developing disease prediction models [19]. Artificial intelligence (AI) is a commonly used term, referring to the development of machines to perform tasks that require intrinsic human abilities such as learning, reasoning, and perception. Machine learning (ML), a subfield of AI, is used to predict unknown information by using algorithms that learn the intrinsic statistical patterns and structures of data. Medical AI is most actively applied in the diagnosis and prediction of prognosis [20]. For example, a previous study attempted to predict placenta accreta spectrum in patients with placenta previa by performing an analysis using ML of MRI-derived texture features and reported their high effects [21]. Another study used various ML algorithms for heart murmur detection based on wavelet transformation [22]. Compared to conventional methods, ML models provide high prediction accuracy and are expected to make significant contributions to diagnosis. In addition, data of various characteristics, which cannot be handled by traditional analysis, can be processed with the ML [23]. Conversely, ML has the disadvantage of requiring massive datasets with high-quality data for training. Another major challenge is the ability to interpret results generated by ML models properly [6].

Studies have been conducted on the use of ML in the field of dentistry to predict toothache [24], impaction of maxillary canine teeth [25], tooth extraction in orthodontic treatment [26], and the presence of root caries [27]. However, as of yet, attempts to use AI in dentistry remain at an elementary stage due to the lack of availability of massive dentistry datasets. Unlike other areas, data in dentistry are often inaccessible for privacy purposes, and the structural complexity renders it difficult to validate [28]. Moreover, the results of using AI in dentistry are sometimes difficult to apply to real-world clinical situations that require highly complex decision-making processes [29]. In particular, it is typically complex and difficult to obtain oral health-related datasets of sufficient quality relative to preschool children, although the KNHANES data include sufficient volume and quality to apply ML. Although the development of an ML-based prediction model for predicting dental caries in preschool children is academically and clinically meaningful, there is a lack of relevant studies. Therefore, in this study, we developed prediction models for ECC by using the national survey data in Korea and evaluated the performance by comparing the ML-based models with a regression model. The proposed prediction models could be the first step towards developing interventions and policies to prevent ECC and can be used in oral health care education.

## 2. Materials and Methods

### 2.1. Research Data and Participants

KNHANES is conducted every three years. Using a two-stage stratified cluster sampling method based on census data, 20 sample households per survey district were selected, and the family members of the sampled households were considered as survey participants. We conducted our study on children from 1 to 5 years of age from the KNHANES 4–7 (2007–2018) survey. Out of 5137 children, 942 children with missing values were excluded, and the data of 4195 children were analyzed.

### 2.2. Variables

Based on responses of surveys, we collected the demographic variables (age, sex, siblings, and household income level), details on oral hygiene management (daily tooth brushing frequency), and maternal details (educational level, birthing age, use of dental floss or interdental brushes, and daily brushing frequency of the mother). Children were classified into three categories: only child, one sibling, and two or more siblings. The mother’s age at the time of giving birth was classified into two categories: under 35, and 35 and above. The mother’s decayed missing filled teeth (DMFT) were selected as an additional maternal variable and classified according to quartiles. As a response variable, we grouped the children based on the number of decayed-filled teeth (dft) into groups of 0 (*n* = 3134) and 1 or more (*n* = 1061). Dentists who participated in the study conducted the oral examination for the DMFT of the mother and dft of the children.

### 2.3. Data Analysis

We performed a chi-square test to examine the distribution of ECC history according to the characteristics of the participants. Analysis of variance (ANOVA) was performed to compare the differences between groups in dft values. To develop and evaluate the prediction models, the entire participant population was divided into training (*n* = 2936) and test (*n* = 1259) datasets with a ratio of 7:3. We conducted a logistic regression analysis with the training datasets to examine the relationship between the characteristics and ECC history of the participants and to develop an ECC prediction model. Moreover, we developed prediction models by implementing various ML algorithms (XGBoost, random forest, and lightGBM). We performed hyperparameter tuning, a process that adjusts an algorithm to improve the accuracy of the prediction model (Supplementary Table S1).

For variable selection, two methods were applied on the training datasets; (1) the variables were selected using backward elimination based on logistic regression with the reference *p*-value of 0.2 [30]; (2) permutation importance was calculated using a random forest classifier, which is more appropriate for non-linear classifiers [31]. For the *k*-fold cross-validation step, we performed 5-fold cross-validation of the model using area under the receiver operator characteristic curve (AUROC) as the measure of model quality. The performance of the model was evaluated with the test dataset using the area under the AUROC, misclassification rate (1-Accuracy), Sensitivity, Specificity, and the Matthews correlations coefficient (MCC) [32,33]. ML algorithms used in our study were developed using open-source Python packages (Scikit-learn, lightgbm: https://lightgbm.readthedocs.io/en/latest/index.html, accessed on 4 August 2021), and XGboost: https://xgboost.readthedocs.io/en/latest/python/index.html, accessed on 4 August 2021). All statistical analyses were performed using SAS (version 9.4; SAS Institute Inc., Cary, NC, USA), with the statistical significance level set at *p* < 0.05.

## 3. Results

Of the children with a dft of 1 or higher, children aged 1, 2, 3, 4, and 5 years accounted for 1.7%, 9.4%, 18.9%, 31.0%, and 38.9%, respectively, of which 54.9% were boys and 45.1% were girls (Table 1). The distribution of children with dft ≥ 1 was higher than those whose mother’s age at childbirth was 35 years or older (*p* < 0.001). In addition, 27.8% of children with a history of dental caries had a mothers’ DMFT score of 7 or higher, whereas 23.6% of mothers whose children never experienced dental caries (dft = 0) had a DMFT of 7 or higher (*p* < 0.01). 

The mean dft of 5 year olds was 1.83 ± 2.78, which was higher than that of 1–4 year olds (Table 2, *p* < 0.001). The mean dft of children according to the mother’s birthing age was 0.70 ± 1.79 for under 35 years and 1.11 ± 2.37 for 35 years and above, which was a statistically significant difference (*p* < 0.001). The mean dft of a child whose mother had 7 or more DMFT was 1.13 ± 2.44, which was significantly higher than that of the other groups (*p* < 0.001).

In order to examine the factors related to ECC history, we selected significant variables from multiple logistic regression analyses using backward elimination in the training dataset. As a result, the final prediction model comprised five characteristics such as age, household income, daily brushing frequency, age of the mother at the time of giving birth, and the mother’s DMFT quartile (Table 3). Based on the final model, the risk of ECC was observed to be low among children who brushed their teeth over three times a day (OR, 0.77; CI, 0.58–0.98) and significantly higher in the group having a maternal DMFT of 7 or higher compared to that having 0 or 1 DMFT (OR, 1.44; CI, 1.11–1.87). Simple logistic regression analysis showed that the risk of ECC was significantly higher in the group where the mother’s birthing age was 35 years or more (OR, 1.60; CI, 1.36–1.89). However, while the OR was higher than 1 in the final prediction model, it was not statistically significant (OR, 1.14; CI, 0.94–1.36).

**Figure 1:**
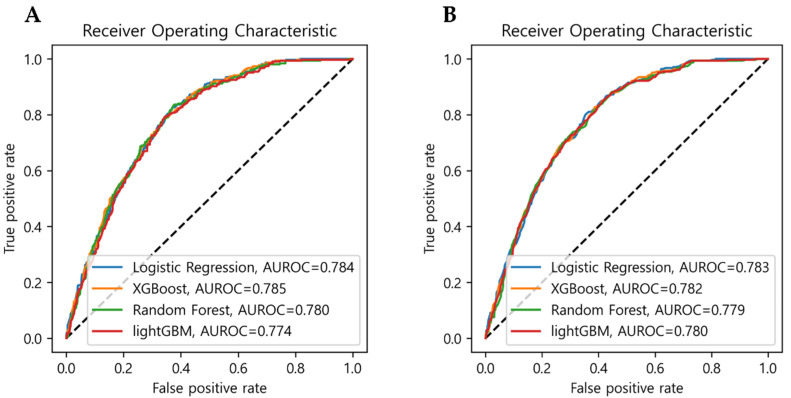
Comparison of the AUROC of the four models. Variables were selected (**A**) using backward elimination based on logistic regression and (**B**) using permutation importance based on random forest.

Among the ECC prediction models comprising five variables selected from logistic regression, the model using XGBoost exhibited the highest AUROC of 0.785, whereas that of the models using logistic regression, a random forest, and lightGBM were 0.784, 0.780, and 0.774, respectively (Figure 1A and Table 4). The logistic regression model exhibited the lowest misclassification rate of 0.235. However, no statistically significant differences were observed between the four models.

We also implemented another variable selection method from a random forest algorithm using permutation importance. As a result, there were four important variables including age, household income, daily brushing frequency, and the mother’s DMFT quartile. In the models including these four variables, the model using the logistic regression exhibited the highest AUROC of 0.783, whereas those of the models using XGBoost, random forest, and lightGBM were 0.782, 0.779, and 0.780, respectively (Figure 1B and Table 4). Similarly, the misclassification rate was the lowest in the logistic regression model. The most models exhibited almost random classification results (MCC < 0.20), except logistic regression which had MCC > 0.20.

## 4. Discussion

We developed ECC prediction models by performing logistic regression analysis on children from 1 to 5 years of age from the 4th–7th KNHANES data (2007–2018) to identify ECC-related factors. The final prediction model considered the age of the child, household income, tooth brushing frequency of the child, mother’s birthing age, and the mother’s DMFT as related variables. It has been widely known that the higher the age, the less frequent brushing, and the lower the economic level of a household, the higher the likelihood of ECC prevalence [34]. Conversely, the sex of the child was excluded from the final prediction model because it had no significant relationship with the prevalence of ECC. A wide variety of conclusions have been reported regarding the relationship between the sex of the child and ECC. While some studies reported that boys are more likely to experience ECC [35], some showed no significant differences due to the sex of the child, which is consistent with the results of this study [36,37]. These differences could be due to the studies conducted at different times and the varied characteristics of the research populations. Furthermore, the number of siblings of a participant was also excluded from the final prediction model as no significance was observed with the prevalence of ECC, as demonstrated in a previous study [37].

Since parent’s knowledge, perception, and behavior on oral health can significantly influence the onset of ECC in children [9,34], we included maternal variables in the analysis. According to the results of the final prediction model, the likelihood of ECC in a child was high if the mother’s DMFT value was high, which is consistent with previous research findings which demonstrated that the oral health status of guardians also affects the oral health of children [9,34]. While the OR value was higher than 1 when the age of the mother at the time of giving birth was 35 years or more, there was no significant association. While a previous study [38] found no significant association between maternal age at childbirth and the prevalence of ECC among children, other studies [39,40] reported a significant relationship between them. In these studies [39,40], the children were divided into three groups based on their mother’s age at their birth (low, middle, and high age). They reported that children in the low maternal age group (22 or 24 and below) had a significantly higher ECC risk than compared to children in the middle and high age groups. However, for the datasets used in our study, the proportion of mothers below 24 years of age at the time of giving birth was extremely low. Therefore, we divided children into two groups according to their mothers’ ages (old age and non-old age), while considering a reference age of high-risk pregnancy (35 years old). We suspect that the relationship between ECC and the mother’s age at the time of giving birth was not significant in our study owing to the differences in the classification criteria. However, the mean dft was significantly higher in children whose mother’s age at birth was 35 years or above than in children whose mother’s age at birth was below 35 years. Furthermore, the education level of mothers was not significantly associated with the ECC history in children. In contrast to our findings, a study conducted in Hong Kong [41] reported a significant relationship between a mother’s low educational level and the high prevalence of ECC in the child. However, a Canadian study [42] showed results consistent with our study. The rise in the average level of education in Korean society in recent years is consistent with our data, which shows that over 80% of the participants had a college degree or higher. Although such upward leveling of educational background can weaken the relationship between the education level and ECC, further research would be required to verify it.

We attempted to reduce the number of variables to conveniently use a prediction model. Compared to the multiple regression model that considered all variables, the AUROC value of our final prediction model changed only 0.002 after removing some variables using the backward elimination method. Therefore, we developed a logistic regression model with five variables as the final prediction model, which showed a higher AUROC than the widely used critical point for prediction model performance (0.7), thereby indicating that the final prediction model was appropriate for predicting ECC in clinical practices. 

In addition, we built prediction models using three ML algorithms that have been widely used in recent disease prediction models and compared their performance with the logistic regression model. As a result, no significant difference was observed in the performance of the ML-based models and the logistic regression model. Considering the limited number of studies conducted by using ML in prediction models in dentistry, it is difficult to compare our study with the existing studies. Although most ML-based prediction models used in the field of medicine demonstrated improved performance compared to traditional statistical methods [43,44,45], not all showed a higher performance than regression models developed in these studies. For example, Sampa et al. [43] developed a blood uric acid prediction model using multiple ML algorithms and reported that the boosted decision tree model showed improved performance compared to the traditional linear regression model. However, among the ML algorithms used in Sampa’s study, the model using a neural network exhibited a lower performance than the linear regression model. This shows that not all ML-based prediction models show higher performance than traditional regression models, and the outcome varies based on the variable characteristics, the database used, and disease characteristics to be predicted. Nevertheless, there is no doubt that ML algorithms such as XGBoost and random forest are very powerful classifiers in many cases [43,44,45].

In this study, two kinds of variable selection methods were applied in the prediction models because selecting variables using only a linear model can bias the results for ML algorithms, which are nonlinear classifiers. Among the methods which are appropriate for nonlinear classifiers, performance importance was used in this study, as suggested by the previous studies [31,45,46]. When the backward elimination based on logistic regression was used, five variables were selected. On the other hand, in the variable selection based on the random forest, only four variables were selected, excluding the mother’s age at childbirth. Regardless of the variable selection method, there was no significant difference in the AUROC values between the regression and the ML-based models, which may mean that the bias due to the variable selection was not significant. The logistic prediction model including four variables also showed similarly good performance compared to the model including five variables (Table 4). However, for the regression model, applying variable selection by a linear model was more appropriate than the nonlinear approach. Therefore, the prediction model ultimately proposed in this study was determined to be a logistic regression model including five variables. 

Owing to the nature of the cross-sectional survey data, although our study examined the association between various variables and ECC, their causal relationship could not be analyzed. In addition, because we only used variables included in the KNHANES data, we did not analyze variables such as feeding behavior, sugar intake, and fluoride use. Furthermore, although KNHANES investigates both paternal and maternal variables, we only examined the maternal variables owing to the high missing frequency in paternal variables, which could result in biased results. Our final prediction model included the maternal DMFT index assessed through a dentist’s examination during the survey, which increases the accuracy of the model due to the accurate measurements. Nevertheless, if a prediction model such as this is to be used in actual clinical practices, the need for a dentist’s examination can be disadvantageous as it reduces the utility of the model. Therefore, further research is required to examine whether the model maintains a high performance when a dentist’s examination is not feasible and is, instead, replaced by a questionnaire. Another drawback of the prediction models developed in this study is their relatively low specificity values. This may be attributed to the skewed distribution of data, which is even worse for the test set [32]. In order to estimate the performance of models, a balance of false positive and negative prediction errors is considered a good characteristic of prediction models [47]. Therefore, further research is needed to develop an ECC prediction model able to overcome this weakness.

Despite several limitations, we believe our proposed prediction model makes a significant contribution to the literature as it can predict ECC in preschool children based on a relatively simple survey and examination. Furthermore, our model was developed using high-quality data that represented the Korean population. Moreover, the model can be used to identify ECC high-risk groups and implement active preventive treatments and to establish policies on ECC prevention as baseline data. We hope to enhance the effects of oral health education in guardians of preschool children by using the proposed model and to contribute to a reduction in ECC prevalence.

## 5. Conclusions

In this study, we developed prediction models using data from the KNHANES (2007–2018) to detect ECC in children from the ages of 1 and 5. According to the final logistic model proposed in this study, the following five variables were found to be risk factors for ECC: children’s age (old), household income (low), teeth brushing frequency (≤1), mother’s birthing age (≥35 years old), and the mother’s DMFT (≥7). The children’s sex and the number of siblings were not associated with the prevalence of ECC. By contrast, their mother’s education level, the use of dental floss or interdental brushes, and the children’s frequency of teeth brushing did not show significant association with the child’s prevalence of ECC. Additionally, we compared the performances of the prediction models and found no evidence of the ML-based models exhibiting a higher predictive performance than the regression model. All four types of prediction models showed AUROC values of 0.7 or higher, which indicated an appropriate level of accuracy for realization in clinical applications. In future research, we are considering undertaking the development of an ECC prediction model with improved performance by using a large-scale longitudinal database or, alternatively, a more recently developed deep-learning algorithm. Furthermore, it is necessary to develop a more practical ECC prediction model recording fewer false positives, which might be more suitable for real-world clinical use.

## Figures and Tables

**Table 1:** Characteristics of participants based on early childhood caries.

Variables		dft ≥ 1		dft = 0		*p*-Value
		*n* = 1061		*n* = 3134		
		*n*	%	*n*	%	
Age of the children	1	18	1.7	817	26.1	<0.001
	2	100	9.4	723	23.1	
	3	201	18.9	628	20.0	
	4	329	31.0	514	16.4	
	5	413	38.9	452	14.4	
Sex	Boy	583	54.9	1620	51.7	0.066
	Girl	478	45.1	1514	48.3	
Children with siblings	Only child	642	60.5	1814	57.9	0.289
	1	396	37.3	1239	39.5	
	≥2	23	2.2	81	2.6	
Household income	Low	63	5.9	141	4.5	0.098
	Low-medium	340	32.0	961	30.7	
	High-medium	381	35.9	1231	39.3	
	High	277	26.1	801	25.6	
Tooth brushing frequency	≤1	165	15.6	828	26.4	<0.001
	2	389	36.7	1036	33.1	
	≥3	507	47.8	1270	40.5	
Education level of the mother	Middle school	13	1.2	43	1.4	0.888
	High school	197	18.6	567	18.1	
	≥College	851	80.2	2524	80.5	
Age of the mother at the time of giving birth	<35	483	45.5	1786	57.0	<0.001
	≥35	578	54.5	1348	43.0	
Use of dental floss or interdental toothbrush of mother	No	473	44.6	1479	47.2	0.141
	Yes	588	55.4	1655	52.8	
Tooth brushing frequency of the mother	≤1	41	3.9	139	4.4	0.070
	2	366	34.5	1189	37.9	
	≥3	654	61.6	1806	57.6	
DMFT of the mother	≤1	224	21.1	833	26.6	0.001
	2–4	346	32.6	1029	32.8	
	5–6	196	18.5	531	16.9	
	≥7	295	27.8	741	23.6	

*p*-value was derived using the chi-square test; dft: decayed or filled primary teeth; DMFT: decayed, missing, or filled permanent teeth.

**Table 2:** Comparison of the dft values based on the characteristics of the participants.

Variables		*n*	Mean	SD	*p*-Value
Age of the children	1	835	0.05	0.47	<0.001
	2	823	0.32	1.20	
	3	829	0.76	1.78	
	4	846	1.44	2.60	
	5	865	1.83	2.78	
Sex	Boy	2203	0.97	2.22	0.011
	Girl	1992	0.81	1.92	
Children with siblings	Only child	2456	0.93	2.15	0.362
	1	1635	0.84	2.00	
	≥2	104	0.76	1.84	
Household income	Low	204	1.20	2.72	0.014
	Low-medium	1301	0.98	2.34	
	High-medium	1612	0.79	1.85	
	High	1078	0.86	1.83	
Tooth brushing frequency	≤1	993	0.59	1.74	<0.001
	2	1425	0.97	2.15	
	≥3	1777	0.99	2.19	
Education level of the mother	Middle school	56	1.14	2.77	0.102
	High school	764	1.02	2.49	
	≥College	3375	0.86	1.97	
Age of the mother at the time of giving birth	<35	2269	0.70	1.79	<0.001
	≥35	1926	1.11	2.37	
Use of dental floss or interdental toothbrush of mother	No	1952	0.88	2.13	0.679
	Yes	2243	0.90	2.05	
Tooth brushing frequency of the mother	≤1	180	1.11	2.82	0.151
	2	1555	0.83	1.94	
	≥3	2460	0.92	2.11	
DMFT of the mother	≤1	1057	0.70	1.81	<0.001
	2–4	1375	0.82	1.89	
	5–6	727	0.96	2.22	
	≥7	1036	1.13	2.44	

*p*-value was derived using ANOVA; dft: decayed or filled primary teeth; DMFT: decayed, missing, or filled permanent teeth.

**Table 3:** Logistic regression results for the prediction of early childhood caries.

Variables		Simple				Multiple				Multiple (Final Model) *			
		Crude OR	95% CI		*p*-Value	Adjusted OR	95% CI		*p*-Value	Adjusted OR	95% CI		*p*-Value
Age of the children	1	1.00				1.00				1.00			
	2	5.25	3.02	9.12	<0.001	5.44	3.12	9.51	<0.001	5.48	3.14	9.58	<0.001
	3	11.38	6.69	19.38	<0.001	12.33	7.16	21.23	<0.001	12.43	7.22	21.40	<0.001
	4	22.87	13.55	38.59	<0.001	25.23	14.74	43.20	<0.001	25.48	14.90	43.59	<0.001
	5	31.40	18.64	52.92	<0.001	33.96	19.81	58.19	<0.001	34.13	19.93	58.46	<0.001
Sex	Boy	1.00				1.00				-			
	Girl	0.82	0.69	0.97	0.018	0.88	0.74	1.06	0.182				
Children with siblings	Only child	1.00				1.00				-			
	1	0.89	0.75	1.06	0.180	0.95	0.78	1.15	0.607				
	≥2	0.92	0.53	1.61	0.766	1.06	0.56	2.00	0.860				
Household income	Low	1.00				1.00				1.00			
	Low-medium	0.74	0.50	1.08	0.517	0.70	0.46	1.09	0.114	0.70	0.45	1.07	0.101
	High-medium	0.65	0.45	0.96	0.024	0.54	0.35	0.84	0.006	0.54	0.35	0.83	0.005
	High	0.74	0.50	1.10	0.622	0.63	0.40	0.98	0.041	0.63	0.41	0.98	0.039
Tooth brushing frequency	≤1	1.00				1.00				1.00			
	2	1.81	1.42	2.30	<0.001	0.99	0.60	1.65	0.973	0.97	0.73	1.27	0.804
	≥3	1.88	1.49	2.37	<0.001	1.06	0.63	1.76	0.836	0.77	0.58	0.98	0.032
Education level of the mother	Middle school	1.00				1.00				-			
	High school	0.66	0.33	1.32	0.234	0.83	0.38	1.78	0.625				
	≥College	0.66	0.34	1.31	0.234	0.86	0.41	1.82	0.696				
Age of the mother at the time of giving birth	<35	1.00				1.00				1.00			
	≥35	1.60	1.36	1.89	<0.001	1.12	0.93	1.35	0.236	1.14	0.94	1.36	0.177
Use of dental floss or interdental toothbrush of mother	No	1.00				1.00				-			
	Yes	1.04	0.88	1.22	0.682	1.05	0.87	1.27	0.585				
Tooth brushing frequency of the mother	≤1	1.00				1.00				-			
	2	1.06	0.68	1.67	0.788	0.99	0.60	1.65	0.809				
	≥3	1.14	0.73	1.77	0.576	1.06	0.63	1.76	0.695				
DMFT of the mother	≤1	1.00				1.00				1.00			
	2–4	1.19	0.95	1.50	0.134	1.21	0.94	1.55	0.132	1.21	0.95	1.55	0.129
	5–6	1.29	0.99	1.68	0.058	1.33	0.99	1.77	0.055	1.34	1.00	1.78	0.048
	≥7	1.40	1.10	1.77	0.006	1.43	1.10	1.86	0.008	1.44	1.11	1.87	0.006
AUROC		-				0.775				0.777			

Analyses were performed with the training dataset (*n* = 2936). * Variables were selected by backward elimination. DMFT, decayed, missing, or filled permanent teeth; AUROC, area under the receiver operating characteristic.

**Table 4:** Summary of the predictive performance of each prediction model.

Model	AUROC	1-Accuracy	Sensitivity	Specificity	MCC
Variable selection by logistic regression using backward elimination *					
Logistic Regression (Final model)	0.784	0.235	0.799	0.531	0.258
XGBoost	0.785	0.237	0.769	0.581	0.148
Random Forest	0.780	0.245	0.759	0.400	0.040
LightGBM	0.774	0.236	0.782	0.546	0.204
Variable Selection by random forest using permutation importance **					
Logistic Regression	0.783	0.232	0.798	0.547	0.260
XGBoost	0.782	0.237	0.770	0.583	0.158
Random Forest	0.779	0.245	0.772	0.480	0.139
LightGBM	0.780	0.239	0.776	0.532	0.174

Analyses were performed with test dataset (*n* = 1259). AUROC: area under the receiver operating characteristic; 1-Accuracy: misclassification rate; MCC: Matthews correlations coefficient. * Five variables were included: age, household income, daily brushing frequency, age of the mother at giving birth, and the mother’s DMFT quartile. ** Four variables were included: age, household income, daily brushing frequency, and the mother’s DMFT quartile.

## Data Availability

The data supporting the findings of this study are available on the KNHANES homepage (https://knhanes.kdca.go.kr/knhanes/main.do (accessed on 25 June 2021)).

## Supplementary Materials

The following are available online at https://www.mdpi.com/article/10.3390/ijerph18168613/s1, Table S1: Summary of the parameter values of each model.
