# Supplementary material for: Prediction Models of Early Childhood Caries Based on Machine Learning Algorithms
Source: Int J Environ Res Public Health. 2021 Aug 15;18(16):8613. doi: 10.3390/ijerph18168613 (PMC8393254; doi:10.3390/ijerph18168613)
Supplement: Supplementary file 1 [file ijerph-18-08613-s001.zip › ijerph-1324461-supplementary.pdf]

Supplemantary Table S1. Summary of the parameter values of each model

Model	Parameter	Value
XGBoost	n_estimators	200
	max_depth	3
	eta	0.02
	gamma	0 (default)
	colsample_bytree	1 (default)
	min_child_weight	1 (default)
	learning_rate	0.01
	Subsample	0.6
random forest	n_estimators	200
	min_samples_split	10
	min_samples_leaf	3
	max_depth	6
lightGBM	n_estimators	200
	learning_rate	0.02
	max_depth	5
	min_child_samples	48
